# Editorial: Brain-inspired navigation and sensing for robots

**DOI:** 10.3389/fnbot.2023.1329324

**Published:** 2023-11-20

**Authors:** Hui Zhao, Fangwen Yu, Xuke Hu, Zhi Xiong, Jianga Shang, Fuqiang Gu

**Affiliations:** ^1^School of Computer Science, China University of Geosciences, Wuhan, China; ^2^College of Computer Science, Chongqing University, Chongqing, China; ^3^Department of Precision Instrument, Tsinghua University, Beijing, China; ^4^Institute of Data Science, German Aerospace Center (DLR), Jena, Germany; ^5^College of Automation Engineering, Nanjing University of Aeronautics and Astronautics, Nanjing, China

**Keywords:** positioning and navigation, brain-inspired navigation, planning, robots, neuromorphic computing, brain-inspired sensing

With the rapid development of artificial intelligence, computer vision, and sensor technologies, robotics have witnessed remarkable progress over the years. However, a significant challenge modern robots have encountered is navigating and interacting with complex and unknown environments. Conventional positioning and sensing methods rely on rigid programming and predefined algorithms, limiting their adaptability and problem-solving capabilities.

Human beings have long drawn inspiration from nature, seeking to replicate its intricate design and exceptional capabilities. In the case of brain-inspired navigation and sensing for robots, researchers have turned to the human brain as a blueprint for creating robots with a heightened ability to interact with the world. These systems enable robots to perceive, process, and respond to their environment in a more human-like manner.

In this editorial, we investigate the significance of brain-inspired navigation and sensing for robots and its transformative potential. Brain-inspired navigation and sensing systems are designed to mimic the sensory and decision-making processes of the human brain. They employ sensors akin to human senses—vision, hearing, touch, and even proprioception—and process this sensory data using advanced machine learning techniques. The outcome is a robot that can interpret its surroundings, adapt to unexpected changes, and respond intelligently, promising increased autonomy and versatility in a wide array of applications.

Articles in this Research Topic offers an overview of the most recent advances and development of brain-inspired positioning, navigation, planning and operation. Hua et al. introduced a positioning system called SmartFPS, which combines wireless and inertial technologies through an end-to-end neural network (a long short-term memory network). SmartFPS takes as input inertial signal sequence and wireless signal sequence, and outputs the current position and direction. To deal with device heterogeneity and improve the positioning accuracy, a generative adversarial network-based transfer learning strategy was proposed. The proposed method was verified by Bluetooth-inertial in a whole floor scenario, and it reports an accuracy of 0.5 m, which outperformed filter-based methods.

Fang et al. explored the use of quantitative stimulus parameters to control the flight of a robo-pigeon in outdoor environments. The authors conducted experiments to determine the impact of different stimulus parameters on the turning angle and turning radius of the robo-pigeon's flight. They found that by adjusting the stimulus parameters (e.g., frequency, duration, inter-stimulus interval), they were able to control the turning radius of the robo-pigeon's flight. The study provides a useful reference for precise turning flight control of the robo-pigeons and has potential applications in the field of robotics and animal behavior studies.

Rivero-Ortega et al. designed a bio-inspired navigation system that can guide a robot to a target location while effectively avoiding both stationary and moving obstacles. The system uses a ring attractor neural network to model the behavior of animals in a group, allowing a robot to navigate in a similar way. The authors compared their approach to the widely-used social force model rapidly exploring random tree star methods and found that it performs well in a variety of scenarios. They suggested that this technology could have applications in areas such as search and rescue, surveillance, and agriculture.

Chao et al. introduced a novel place cell-based path planning algorithm for drones, which uses a spiking neural network to create efficient routes. It draws the concept of propagating waves in a cognitive map to find feasible and collision-free path. Experiments were conducted by simulation in Airsim, and results justified the validity of the proposed method. It is also more biologically plausible and might have higher computational efficiency when implemented on neuromorphic hardware.

Yan et al. proposed a method for enabling multiple robots to cooperatively explore and navigate through various terrestrial environments. To deal with the exploration failure in challenging environments, a self-healing method was proposed, which allows other robots to re-allocate the failed robot's tasks and continue the exploration task. Experimental results obtained by simulation showed the effectiveness of the proposed method.

While the potential of brain-inspired navigation and sensing for robots is immense, it also brings forth challenges and ethical considerations. As robots become more autonomous and intelligent, issues related to privacy, safety, and job displacement must be addressed. It is essential that we proceed with careful consideration of the societal implications of these technologies.

In conclusion, brain-inspired navigation and sensing is ushering in a new era for robotics. The ability to replicate and enhance human-like sensory perception and problem-solving capabilities in robots is a testament to the remarkable progress in the field. These robots are becoming invaluable tools for various industries, and their applications are limited only by our imagination. However, as with any transformative technology, it is our responsibility to ensure its ethical and responsible use. Brain-inspired robotics has the potential to improve our lives in ways we cannot yet fully comprehend, and it is up to us to harness this potential for the benefit of society as a whole.

## Author contributions

HZ: Writing—original draft. FY: Writing—review & editing. XH: Writing—review & editing. ZX: Writing—review & editing. JS: Writing—review & editing. FG: Writing—review & editing.

